# Disease Severity in Moderate-to-Severe COVID-19 Is Associated With Platelet Hyperreactivity and Innate Immune Activation

**DOI:** 10.3389/fimmu.2022.844701

**Published:** 2022-03-11

**Authors:** Kai Jakobs, Leander Reinshagen, Marianna Puccini, Julian Friebel, Anne-Christin Beatrice Wilde, Ayman Alsheik, Andi Rroku, Ulf Landmesser, Arash Haghikia, Nicolle Kränkel, Ursula Rauch-Kröhnert

**Affiliations:** ^1^Department of Cardiology, Charité University Medicine Berlin, Campus Benjamin Franklin, Berlin, Germany; ^2^German Centre for Cardiovascular Research (DZHK), Partner Site Berlin, Berlin, Germany; ^3^Berlin Institute of Health (BIH), Berlin, Germany; ^4^Department of Hepatology and Gastroenterology, Charité University Medicine Berlin, Campus Virchow, Berlin, Germany

**Keywords:** COVID-19, platelet hyperactivity, immunothrombosis, inflammation, platelet-leucocyte aggregates, disease severity, survival

## Abstract

**Background:**

Hemostasis and inflammation are both dysregulated in patients with moderate-to-severe coronavirus disease 2019 (COVID-19). Yet, both processes can also be disturbed in patients with other respiratory diseases, and the interactions between coagulation, inflammation, and disease severity specific to COVID-19 are still vague.

**Methods:**

Hospitalized patients with acute respiratory symptoms and with severe acute respiratory syndrome coronavirus 2 (SARS-CoV2)-positive (COV^pos^) and SARS-CoV2-negative (COV^neg^) status were included. We assessed adenosine diphosphate (ADP)-, thrombin receptor activator peptide 6 (TRAP)-, and arachidonic acid (AA)-induced platelet reactivity by impedance aggregometry, as well as leukocyte subtype spectrum and platelet-leukocyte aggregates by flow cytometry and inflammatory cytokines by cytometric bead array.

**Results:**

ADP-, TRAP-, and AA-induced platelet reactivity was significantly higher in COV^pos^ than in COV^neg^ patients. Disease severity, assessed by sequential organ failure assessment (SOFA) score, was higher in COV^pos^ than in COV^neg^ patients and again higher in deceased COV^pos^ patients than in surviving COV^pos^. The SOFA score correlated significantly with the mean platelet volume and TRAP-induced platelet aggregability. A larger percentage of classical and intermediate monocytes, and of CD4^pos^ T cells (T_H_) aggregated with platelets in COV^pos^ than in COV^neg^ patients. Interleukin (IL)-1 receptor antagonist (RA) and IL-6 levels were higher in COV^pos^ than in COV^neg^ patients and again higher in deceased COV^pos^ patients than in surviving COV^pos^. IL-1RA and IL-6 levels correlated with the SOFA score in COV^pos^ but not in COV^neg^ patients. In both respiratory disease groups, absolute levels of B-cell-platelet aggregates and NK-cell-platelet aggregates were correlated with *ex vivo* platelet aggegation upon stimulation with AA and ADP, respectively, indicating a universal, but not a COVID-19-specific mechanism.

**Conclusion:**

In moderate-to-severe COVID-19, but not in other respiratory diseases, disease severity was associated with platelet hyperreactivity and a typical inflammatory signature. In addition to a severe inflammatory response, platelet hyperreactivity associated to a worse clinical outcome in patients with COVID-19, pointing to the importance of antithrombotic therapy for reducing disease severity.

**Graphical Abstract:**
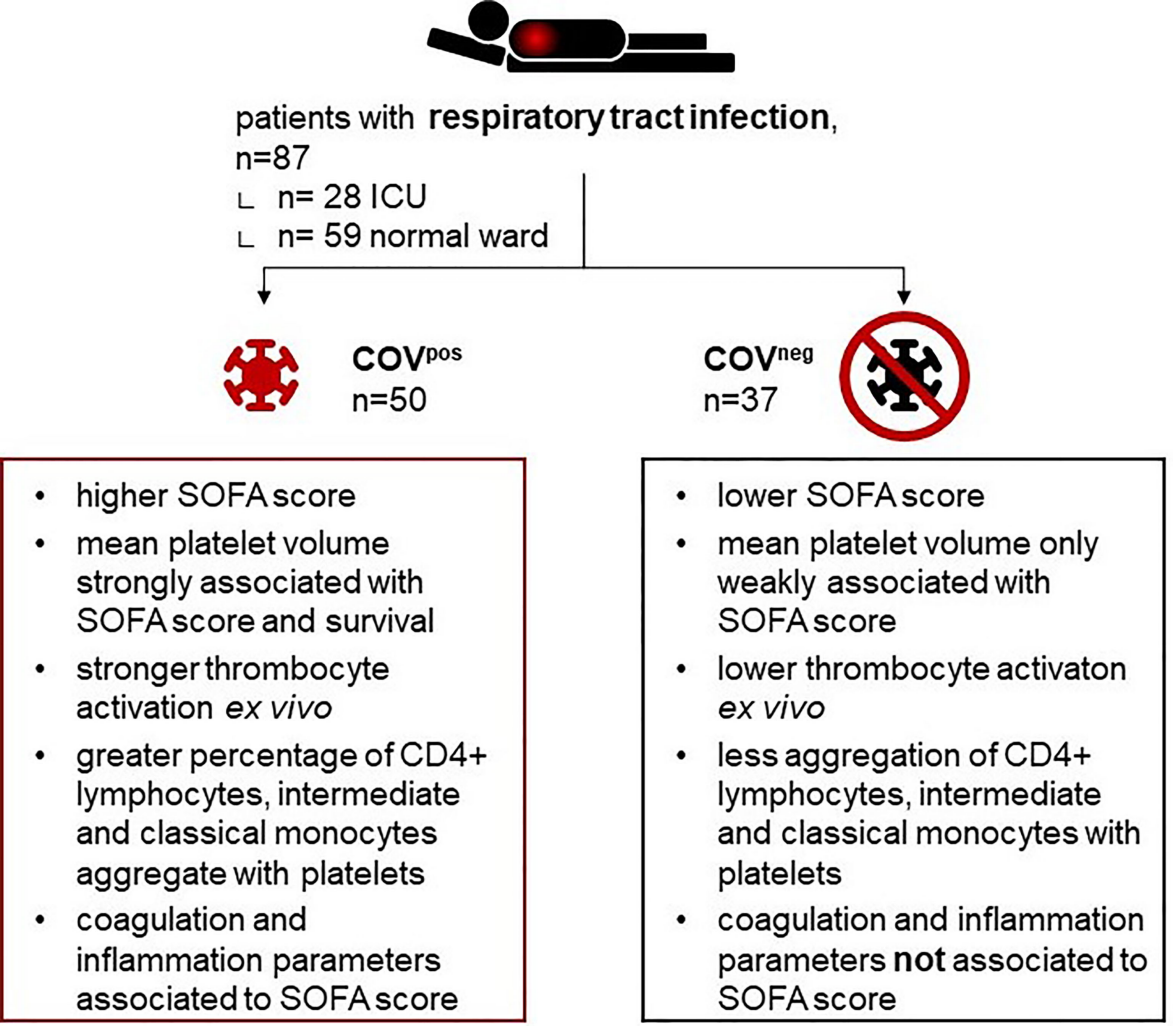
Depicting study design and main observations.

## Introduction

The coronavirus disease 2019 (COVID-19) pandemic, caused by severe acute respiratory syndrome coronavirus 2 (SARS-CoV2), continues to affect humans all around the world (1). While vaccinations have greatly helped to lower the number of patients admitted to intensive care with severe COVID-19, newly emerging SARS-CoV-2 variants and refusal to receive vaccination in some countries still heavily fill ICU wards (2).

Acute dysregulations in hemostasis and inflammation are prominent features of patients with moderate-to-severe COVID-19 (3). Thrombi have been detected in the lung, heart, brain, and liver of COVID-19 patients, and the prevalence of deep vein thrombosis in hospitalized patients with an infection is significantly increased (4–6). Microthrombosis, also extensively documented by autopsy reports, reflects the increased platelet activation and subsequent fibrin clot formation in the pulmonary microvasculature in 80%–100% of lungs examined (7). The increased risk of thromboembolic events observed during moderate-to-severe COVID-19 is associated with the increased morbidity and mortality of these patients (8, 9).

Upon stimulation with thrombin, platelets from patients with COVID-19 released more interleukin (IL)-1β and soluble cluster of differentiation 40 ligand (sCD40L) than platelets of healthy controls (4). Moreover, lower amounts of thrombin were required for platelets from COVID-19 patients to achieve aggregation than for platelets from healthy controls, further suggesting that these platelets have an increased hyperactive potential, contributing to the overall inflammation often observed during the infection with SARS-CoV2 (10). Platelet-specific granule content, including platelet factor 4 (PF4) and serotonin was significantly elevated in the plasma of patients with COVID-19 (11, 12).

Crosstalk between platelets and the immune system involves a variety of mechanisms (13, 14). Beyond paracrine mechanisms, platelets can also form aggregates with various leukocyte subtypes, such as neutrophils, monocytes, and T cells (11, 14, 15). Platelet-leukocyte aggregates have been suggested to drive vascular disease and may potentially represent a biomarker for thrombotic events (9, 16).

Increased levels of IL-6 and C-reactive protein (CRP) are associated with a worse outcome from COVID-19, suggesting that inflammation contributes as a critical mediator to the heightened mortality of those patients (17, 18).

The actual processes governing interactions between platelets, coagulation, and inflammation in COVID-19 are still not well known. In particular, knowledge about distinct immuno-thrombotic pathways in COVID-19 which may differ from other infectious respiratory diseases is limited. Importantly, subjects without acute respiratory symptoms, such as healthy persons or patients without any respiratory symptoms have been chosen as control groups in many clinical studies (15, 19–21). While these comparisons give valuable first insight, similar mechanisms might be active in COVID-19 and in other respiratory diseases (22). Thus, there is still a lack of knowledge about the typical features that characterize the patients with acute respiratory syndromes caused by COVID-19 as compared with those with non-COVID-19-associated acute respiratory infections.

## Material and Methods

### Study Design and Subjects

All patients that were included into this study were admitted to our clinic due to acute respiratory infectious disease. COV^pos^ had to be SARS-CoV2 positive confirmed by polymerase chain reaction (PCR) testing. COV^neg^ suffered from pneumonia or infect-triggered acute exacerbation of COPD and had to be SARS-CoV2 negative confirmed by PCR. Individuals had to be at least 18 years old and did not suffer from a known hematological or hemostatic disease, coagulopathy, or acute bleeding event. Patients form ICU or normal floor were eligible. Dual antiplatelet therapy was prohibited. Patients were recruited between May 2020 and May 2021. Routinely clinically assessed blood values were determined by the hospital laboratory (Labor Berlin, Berlin, Germany). Mean platelet volume is assessed by the routine diagnostics lab by impedance-based particle counting. Within the same measurement, counts and size of platelets, as well as erythrocytes and leukocytes, are assessed in diluted samples and without lysis. Leukocyte counts are assessed in a separate measurement after erythrocyte lysis. The study was approved by the local ethics committee (EA2/066/20, EA4/147/15). The study was conducted in compliance with the 1964 Declaration of Helsinki and its amendments and the Principles of Good Clinical Practice by the International Council for Harmonization 1996.

### Blood Sampling

Blood was drawn from the cubital veins using ethylenediaminetetraacetic acid (EDTA) (3 ml, Vacurette^®^, Greiner Bio-One, Kremsmünster, Austria), citrate (3 ml, 3.2% sodium citrate, Vacurette^®^, Greiner Bio-One, Kremsmünster, Austria), and hirudin tubes (Sarstedt-Monovette^®^, Sarstedt, Nümbrecht, Germany). Whole blood was separated for the experiments requiring plasma by centrifugation (1,200×*g*, 10 min, room temperature) and stored at −80° for further analysis.

### Multiple Electrode Aggregometry

In hirudinized whole blood which was previously diluted with 0.9% sodium chloride, platelet’s reactivity to TRAP (32 μmol/l), ADP (6.4 μmol/l), and AA (0.5 mmol/l) was measured by multiple electrode aggregometry (MEA; Multiplate^®^ Analyzer; Roche, Germany; regents also by Roche) not later than 3 h after sampling. According to the manufacturers’ instructions, a measurement time of 6 min was set, and the area under the curve was calculated and translated to Multiplate^®^-specific units. The exact method was reported previously (23, 24).

### Flow Cytometric Quantification of Leukocytes and Platelet-Leukocyte Aggregates

Flow cytometric characterization of leukocytes was performed as previously established (25). Within 1 h of collection, 100 µl of EDTA-anticoagulated whole blood was added to a master mix consisting of 100 µl fluorescence-activated cell scanning (FACS) staining buffer (BioLegend, San Diego, CA, USA) and 2 µl of each of the following antibodies: anti-human CD14-Pacific Blue™, CD16-Brilliant Violet 510™, CD4-Brilliant Violet 605™, CD45-Brilliant Violet 711™, CD3-Alexa Fluor 488™, CD26-PE, CD19-PE/Dazzle 594™, CD8-PE/Cyanine7, and CD41-Alexa Fluor 647™ (all BioLegend, San Diego, CA, USA). The mixture was incubated in the dark at room temperature for 30 min. Stained samples were fixed by adding 800 µl of 0.5% paraformaldehyde in phosphate-buffered saline (Sigma-Aldrich, St. Louis, MO, USA) and kept at +4°C in the dark. Samples were acquired on an Attune NxT Acoustic Focusing Cytometer (ThermoFisherScientific, Waltham, MA, USA) within 3 days. Stability of signal detection was verified and documented by daily measurements of Attune Performance tracking beads. Kaluza version 2.1 software (Beckman Coulter, Brea, CA, USA) was used for gating. Scatter parameters were interpreted as measures of morphological features, i.e., side scatter as a measure of granularity and forward scatter as a measure of cell size.

### Cytokine Measurements

The cytokines IL-1RA, IL-2, IL-6, IL-7, IL-10, monocyte chemotactic protein 1 (MCP-1), chemokine (C-C motif) ligand (CCL) 3, chemokine (C-X-C Motif) ligand (CXCL) 8, CXCL10, interferon (IFN)-α2, IFN-γ, granulocyte colony-stimulating factor (GCSF), and tumor necrosis factor alpha (TNF-α) were determined in platelet-depleted plasma samples using the “COVID-19 Cytokine Storm Panel 1” 13-plex bead array (BioLegend, San Diego, CA, USA) according to the manufacturer’s instructions. Data were acquired on the flow cytometer mentioned above and analyzed using Kaluza version 2.1 software.

### ELISA

Thrombin-antithrombin complex (TAT) ELISA (AssayPro, St. Charles, MO, USA) was performed according to manufacturer’s instructions and measured using the plate reader Tecan Infinite 200Pro (Tecan Group, Maennedorf, Switzerland).

### Statistics

All reported probability values are two sided, and a value of *p* < 0.05 was considered statistically significant. Median values and quartiles are reported, and nonparametric tests were used if not stated otherwise. If not stated differently, Mann–Whitney *U* test or Chi-squared test were used to evaluate differences between two groups. Correlations were calculated with Spearman’s test. For network building *p* < 0.01 and *r* > 0.3 or *r* < −0.3 were used as cutoffs for showing connections. Statistics were calculated using SPSS Statistics, version 27 for Windows and macOS (IBM, Armonk, NY, USA) and R version 4.1.0 (2021-05-18). Graphs and networks were plotted using R within R Studio version 1.3.1093 (RStudio PBC, Boston, MA, USA).

## Results

### Patients’ Characteristics

More patients in the COV^neg^ group had chronic obstructive lung disease (**Table 1**). More patients had received prophylactic and less received intermediate-dose anticoagulation in the COV^neg^ than COV^pos^ cohort (**Table 2**). There was no difference in the frequency of patients receiving therapeutic dose anticoagulation (**Table 2**). Administration of oral glucocorticoids and inhalative bronchodilators was more common in COV^pos^ (**Table 2**). In COV^pos^, 10 out of 50 individuals died compared with none in COV^neg^ patients. The sequential organ failure assessment (SOFA) score was calculated for all patients with sufficient data (COV^pos^ *n* = 33, COV^neg^ *n* = 21). Laboratory parameters did not differ significantly between COV^neg^ and COV^pos^ patients (**Table 3**). Four COV^pos^ patients and two COV^neg^ patients had thrombocytopenia as defined by <150 thrombocytes/nL. No patient participating on our study had received vaccination against SARS-CoV2 prior to study participation.

**Table 1:** Patient characteristics—demographics and preexisting conditions.

	COV^neg^ (*n* = 37)	COV^pos^ (*n* = 50)	Mann–Whitney *U* or Chi-squared test
**Demographics**			
Age (years)	73 (58; 81)	69 (54.8; 76.5)	0.078
Men (% per group)	57%	70%	0.259
BMI (kg/m^2^)	25.1 (23.2; 29.3)	28.7 (24.7; 76.5)	0.1030
Patients on ICU (% per group)	22%	40%	0.104
Respiratory rate (breaths per minute)	18 (16.25; 19)	18.7 (18; 21.3)	0.078
Heart rate (beats per minute)	77 (70; 88.5)	81.5 (72.8; 94.8)	0.259
Systolic blood pressure (mmHg)	131 (116; 149.5)	123.5 (110; 140)	0.103
Diastolic blood pressure (mmHg)	75 (66; 85.5)	70 (60; 80)	0.141
**Preexisting conditions**			
Coronary artery disease (% per group)	32.4%	14%	0.065
Arterial hypertension (% per group)	67.6%	62%	0.655
Diabetes (% per group)	48.6%	58%	0.632
Dyslipidemia (% per group)	29.7%	28%	1
COPD (% per group)	35.1%	6%	0.001

Data about demographics and preexisting conditions of COV^pos^ and COV^neg^ absolute numbers or median values with quartiles are shown.

**Table 2:** Patient characteristics—concomitant medication.

	COV^neg^ (*n* = 37)	COV^pos^ (*n* = 50)	Mann–Whitney *U* or Chi-squared test
Acetylsalicylic acid (% per group)	32.4%	36%	0.821
Clopidogrel (% per group)	0%	4%	0.505
Prophylactic anticoagulation (% per group)	56.8%	24%	0.003
Intermediate dose anticoagulation (% per group)	8.1%	32%	0.009
Therapeutic dose anticoagulation (% per group)	35.1%	44%	0.51
Statins (% per group)	24.3%	24%	1
ACE blocker (% per group)	35.1%	24%	0.339
Angiotensin II receptor blocker (% per group)	16.2%	22%	0.591
Beta blocker (% per group)	46%	28%	0.113
Aldosterone antagonist (% per group)	13.5%	6%	0.277
Diuretics (% per group)	50%	38%	0.383
Oral glucocorticoids (% per group)	19%	56%	0.001
Remdesivir (% per group)	0%	2%	1
Tocilizumab (% per group)	0%	2%	1
Inhalative bronchodilators (% per group)	59.5%	84%	0.014

Data about concomitant medication of COV^pos^ and COV^neg^ in absolute numbers are shown.

**Table 3:** Patient characteristics—laboratory values.

	COV^neg^ (*n* = 37)	COV^pos^ (*n* = 50)	Mann–Whitney *U* or Chi-squared test
Creatinine (mg/dl)	0.94 (0.79; 1.3)	0.90 (0.66; 1.16)	0.354
Urea (mg/dl)	34 (24.5; 52.5)	48 (27.3; 64.5)	0.164
NT-proBNP (ng/l)	468 (251; 2318)	498 (125; 1834)	0.499
CRP (mg/dl)	62.1 (37.7; 103.5)	69.8 (18.9; 126.9)	0.880
Hemoglobin (g/dl)	11.7 (10.1; 13.4)	10.9 (9.3; 21.4)	0.140
Leukocytes (n/nl)	9.3 (6.7; 11.8)	8.5 (6.8; 12.6)	0.837
Lymphocytes (n/nl)	1.22 (0.87; 1.96)	1.06 (0.74; 1.50)	0.214
Lymphocytes (% of leukocytes)	12 (9.7; 23.2)	13.2 (8; 18.1)	0.508
Thrombocytes (n/pl)	279 (209; 321)	300.5 (247; 397)	0.057
Mean platelet volume (fl)	10.3 (9.7; 10.9)	10.4 (9.8; 11.6)	0.269
INR	1.07 (1.00; 1.25)	1.12 (1.05; 1.21)	0.307
aPPT (s)	36.4 (31.4; 43.3)	38.8 (32.8; 47.5)	0.435

Data about laboratory values of COV^pos^ and COV^neg^ in median values with quartiles are shown.

### Higher Platelet Reactivity in Patients With COVID-19 Is Associated With Disease Severity and Death

**Figure 1:**
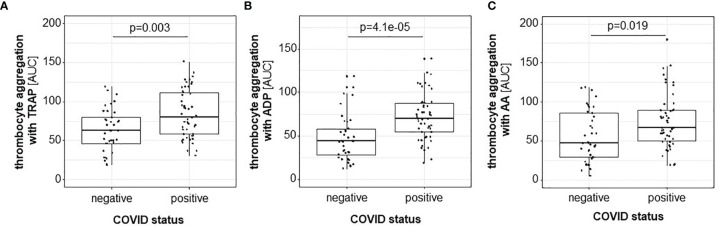
Platelet aggregation induced by TRAP **(A)**, ADP **(B)**, and AA **(C)**, as assessed by MEA, is higher in COV^pos^ (*n* = 50) than in COV^neg^ (*n* = 37) patients.

Platelets from COV^pos^ patients exhibited higher TRAP-induced aggregability than platelets from COV^neg^ patients (**Figure 1A**). As TRAP is a strong coagulation stimulus, this observation points to the hyperaggregability of platelets under stimulation with a thrombin-substitute in COVID-19. ADP-induced platelet aggregability was also increased COV^pos^ as compared with COV^neg^ patients (**Figure 1B**), although ADP is a much weaker platelet agonist than TRAP. Additionally, also AA-induced platelet aggregation was higher in COV^pos^ than COV^neg^ patients (**Figure 1C**). No significant differences between COV^pos^ and COV^neg^ patients were observed for platelet counts or MPV (**Supplemental Figure 1**).

**Figure 2:**
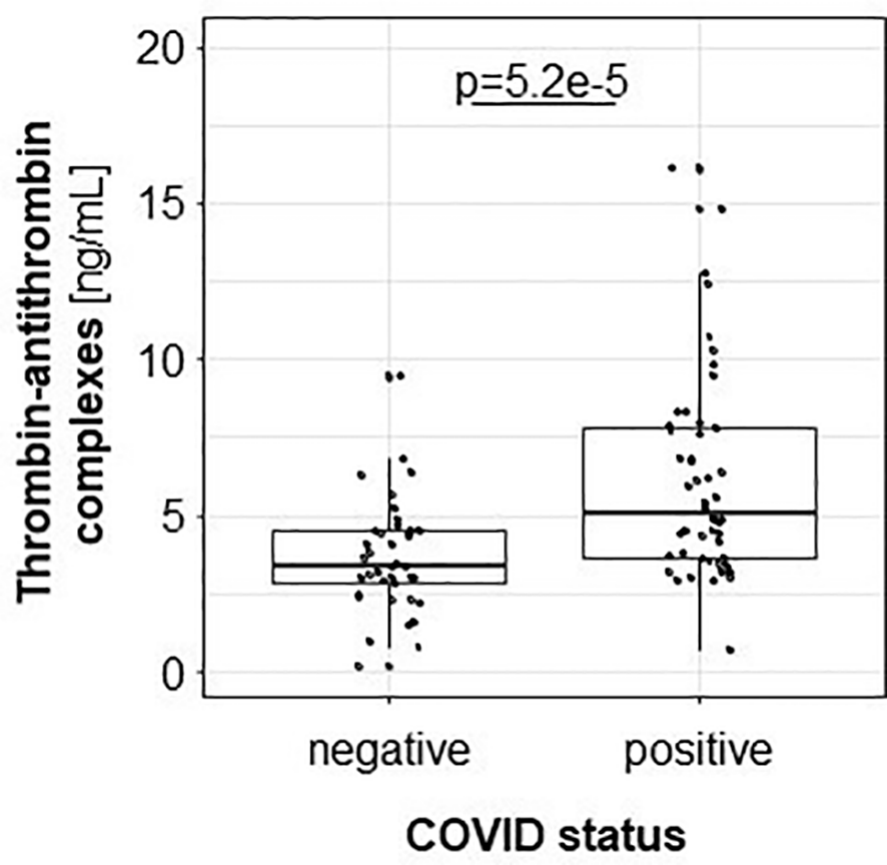
Thrombin-antithrombin complex is higher in COV^pos^ (*n* = 50) than in COV^neg^ (*n* = 37).

Since TAT is a surrogate parameter for thrombin generation and thrombin is the strongest known platelet activator (26), TAT was quantified in both patient groups. Higher TAT levels were measured in COV^pos^ than COV^neg^ patients, reflecting the linkage between higher platelet reactivity and activated coagulation system (**Figure 2**).

**Figure 3:**
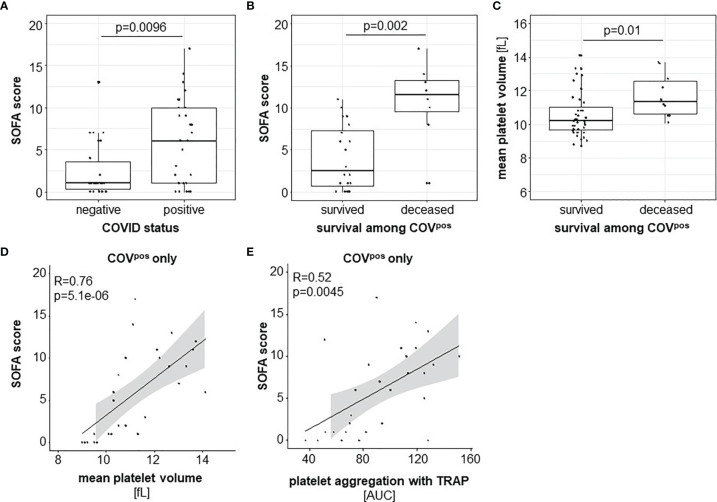
Disease severity indicated by the SOFA score was higher in COV^pos^ (*n* = 50) than in COV^neg^ (*n* = 37) patients **(A)** and higher among COV^non-surv^ (*n* = 10) than COV^surv^ (*n* = 40) COV^pos^ patients **(B)**. MPV was higher in COV^non-surv^ than in COV^surv^ COV^pos^ patients **(C)**. Within COV^pos^, SOFA score correlated with MPV **(D)** and TRAP-initiated platelet activation **(E)**.

In our study, SOFA score values differed significantly between COV^pos^ and COV^neg^ (**Figure 3A**) and between COV^surv^ and COV^non-surv^ patients (**Figure 3B**). MPV was higher in COV^non-surv^ compared with COV^surv^ patients (**Figure 3C**). MPV- (**Figure 3D**) and TRAP-6-induced platelet aggregation (**Figure 3E**) correlated positively with the SOFA score values only in COV^pos^ but not in COV^neg^ patients. These findings point to an important role of platelet function for the clinical prognosis of patients suffering from COVID-19. Instead, levels of individual leukocyte subtype aggregates with platelets did not correlate with SOFA score in COV^pos^ or in COV^neg^ patients.

### Inflammation Characteristics Related to COVID-19 and Clinical Outcome

**Figure 4:**
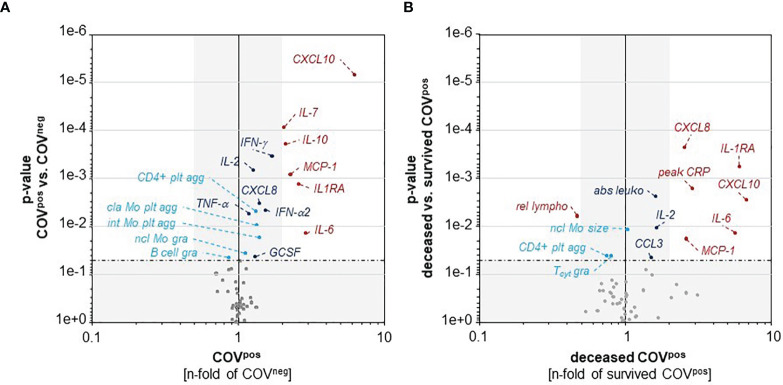
CXCL10, IL-6, MCP-1, and IL-1RA are higher in COV^pos^ than in COV^neg^ patients **(A)** and also higher in deceased than in surviving COV^pos^ patients **(B)**. A higher percentage of classical and intermediate monocytes and CD4^pos^ T_H_ lymphocytes form aggregates with platelets in COV^pos^ than in COV^neg^ patients **(A)**. Deceased COV^pos^ patients showed lower relative abundance of lymphocytes and higher total leukocyte counts than surviving COV^pos^ patients **(B)**. *p*-value of 0.05 is indicated by the horizontal dashed-dotted line. *p*-values higher than 0.05 and less-than-twofold changes between groups are underlaid in grey. plt agg, platelet aggregates; gra, granularity; cla Mo, classical monocytes; int Mo, intermediate monocytes; ncl Mo, nonlassical monocytes.

We observed more than 2-fold higher plasma levels of IL-6, IL-7, IL-10, IL-1RA, MCP1, and CXCL10 in COV^pos^ as compared with COV^neg^ patients, pointing to the severe cytokine burst (**Figure 4A**). The cytokines IL-2, CXCL8, IFN-α2, IFN-γ, GCSF, and TNF-α were less than 2-fold higher in COV^pos^ in comparison with COV^neg^ patients (**Figure 4A**). Moreover, a higher percentage of CD4^pos^ T_H_ lymphocytes, CD14^hi^CD16^neg^ classical monocytes, and CD14^hi^CD16^pos^ intermediate monocytes formed aggregates with platelets in COV^pos^ as compared with COV^neg^ patients (**Figure 4A** and **Supplemental Figure 2**). The granularity of CD14^lo^CD16^pos^ nonclassical monocytes was higher and the granularity of CD19^pos^ B lymphocytes was lower in COV^pos^ in comparison with COV^neg^ (**Figure 4A** and **Supplemental Figure 2**), potentially indicating a differential activation state of these cell types.

Within the COV^pos^ group, we also compared our panel of inflammatory parameters between COV^surv^ and COV^non-surv^ patients (**Figure 4B**). Values of IL-6, IL-1RA, CXCL8, MCP1, and CXCL10 were over 2-fold higher in COV^non-surv^ than in COV^surv^. Moreover, lower relative lymphocyte abundance and higher absolute leukocyte count, IL-2, and CCL3 levels were observed in the COV^non-surv^ versus COV^surv^ patients. In COV^non-surv^ patients, the granularity of CD8^pos^ cytotoxic T cells as well as the percentage of T_H_ cells forming aggregates with platelets were lower than in COV^surv^ patients.

### SOFA Score Correlated With Typical Markers of Platelet Reactivity and Inflammation in COVID-19 Only

**Figure 5:**
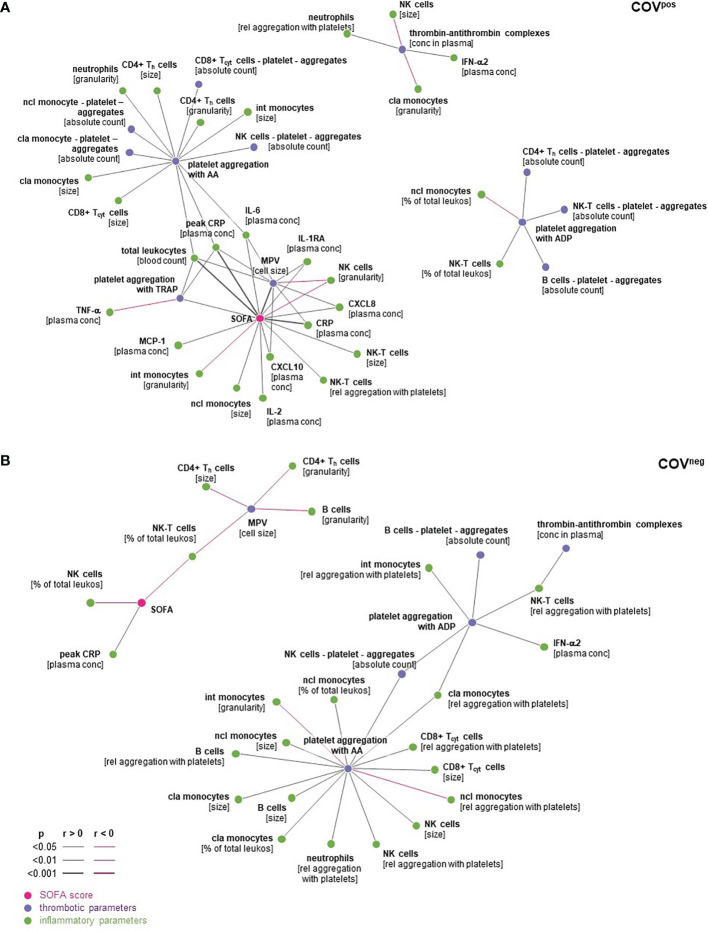
Spearman’s correlations in COV^pos^ **(A)** and COV^neg^ **(B)** were expressed as dark grey lines if positively correlated and pink lines if correlated inversely. Correlations with *p* < 0.05 are depicted. cla Mo, classical monocytes; int Mo, intermediate monocytes; ncl Mo, nonclassical monocytes; T_h_, CD4^pos^ helper T cells; T_cyt_, CD8^pos^ cytoxic T cells; NK-T, natural killer T cells; NK cells, natural killer cells; peak CRP, highest CPR level during hospital stay.

Correlations between the SOFA score, parameters of platelet function, and inflammation are visualized by networks for patients with COVID-19 (**Figure 5A**), with other respiratory disease (**Figure 5B**), and for parameters correlated in both cohorts to elucidate common effects (**Supplemental Figure 3**).

In COV^pos^ patients, the SOFA score correlated with CRP at the time of blood sampling, peak CRP level during hospital stay, leukocyte count, and MPV- and TRAP-induced platelet aggregation. Peak CRP level during hospital stay was strongly correlated with platelet aggregation after stimulation with TRAP-6 and AA (**Figure 5A**). Moreover, IL-6 was correlated with MPV, platelet aggregation after stimulation with TRAP-6 and AA in COV^pos^ but not in COV^neg^ patients (**Figures 5A, B**). In contrast to COV^pos^, only peak CRP but no platelet function marker correlated with the SOFA score in COV^neg^ patients (**Figure 5B**). Correlation between SOFA and peak CRP was much stronger in COV^pos^ than in the COV^neg^ group (**Supplemental Figure 3**). These findings support a link between characteristic markers of inflammation and platelet reactivity and SOFA score for patients with COVID-19 but not for those with other acute respiratory diseases. This panel of characteristic markers is associated with the clinical outcome in COVID-19.

In both patient groups with respiratory symptoms, platelet aggregation upon stimulation with ADP or with AA correlates with the absolute number of platelet aggregates formed with B lymphocytes and with NK cells, respectively (**Supplemental Figure 3**).

## Discussion

The central findings of our study are as follows:

- The platelet reactivity is higher in COV^pos^ than in COV^neg^ patients with an acute respiratory disease and is associated with higher mortality in COV^pos^ patients.
- A higher percentage of T_H_ lymphocytes and classical and intermediate monocytes form aggregates with platelets in COV^pos^ than in COV^neg^ patients.
- The SOFA score as a measure for the clinical outcome strongly correlates with markers of platelet hyperreactivity, CRP, and leukocyte count in COV^pos^ but not in COV^neg^ patients.

These findings suggest a relation between increased markers of platelet reactivity, inflammation, and the clinical outcome for patients with COVID-19 but not for those with other acute respiratory diseases.

### Higher Platelet Reactivity Relates to Survival in Patients With COVID-19

We demonstrated higher platelet reactivity measured by MEA in COVID-19 compared with patients with other acute respiratory disease. Less ADP- and TRAP-induced aggregation (20) and no significant differences for TRAP- and AA-induced aggregation compared with healthy volunteers or reference ranges (19) have previously been reported in patients hospitalized or receiving ICU-level care. Another study demonstrated a predictive value of ADP- and TRAP-induced platelet activation for the duration of the hospital stay (20). However, in these studies, COVID-19 patients were compared only with healthy controls but not with diseased individuals. In our study, platelets of patients with COVID-19 reacted stronger to TRAP, ADP, and AA compared with those patients suffering from other pulmonary infections. In linkage between coagulation system and platelet hyperreactivity, we found TAT to be higher in COV^pos^ than in COV^neg^ patients. Elevated thrombin level may explain the increased platelet reactivity as well as the higher numbers of platelet-leukocyte complexes despite antiplatelet therapy in patients with COVID-19.

Comparing COV^surv^ and COV^non-surv^ groups, we observed a higher MPV in COV^non-surv^, pointing to a hyperreactivity of platelets *in vivo* (27). A retrospective analysis including patients hospitalized for COVID-19 also suggested that MPV relates to the clinical outcome (28). The SOFA score values predict disease severity and mortality in patients with COVID-19 (29, 30). Our data additionally link TRAP-induced platelet aggregation and MPV as markers of platelet reactivity with SOFA score. This once again stresses the important relation between hyperreactive platelets and disease severity.

In patients with COVID-19, thrombocytopenia has been shown to be associated with a worse clinical outcome (31). In our patients, we found four COV^pos^ and two COV^neg^ individuals presenting thrombocytopenia at the time point of platelet function measurement, reflecting the disease severity of our patient cohort.

### Cytokine Signature in COV^pos^ Compared with COV^neg^

Cytokines typically associated with COVID-19-induced “cytokine storm” were mostly higher in COV^pos^ compared with COV^neg^, verifying earlier reports and also in comparison with patients with non-COVID-19 respiratory diseases (32–34). Regarding disease severity, we observed higher levels of IL-6, IL-1RA, MCP1, CXCL8, and CXCL10 comparing COVID survivors and nonsurvivors. MCP1 and CXCL10 as chemotactic agents for monocytes and CXCL8 and CXCL10 as monocyte-derived cytokines point to the central role of monocytes in this setting (35). IL-6 as cytokine amplifier is known to play a prognostic role in COVID-19 (35). IL-1RA antagonizes IL-1 and can also be derived from monocytes (36). Plasma values of IL-1RA correlate with a worse clinical prognosis in COVID-19 (36). Within our COVID cohort, nonsurvivors were characterized by higher cytokine levels, especially IL-1RA, IL-6, MCP-1, CXCL8, and CXCL10 than patients who survived COVID-19.

### Differential Leukocyte-Platelet Aggregate Formation in COV^pos^ Compared With COV^neg^

In COVID-19, monocytes have been shown to release procoagulant proteins in a platelet-dependent manner (22). In line with other studies, we here demonstrate differences in monocyte-platelet conjugates in comparison with patients with acute respiratory diseases other than COVID-19. We found a higher proportion of classical and intermediate monocyte-platelet aggregates in COV^pos^ compared with COV^neg^. These findings are in line with data of other studies, which also demonstrated more platelet aggregates with monocytes in patients with COVID-19 compared with healthy individuals (15, 37) or not further specified patients as controls (22). This points to the importance of the innate immune system interacting with platelets in COVID-19.

In this study, more T_H_ lymphocytes formed aggregates with platelets in COV^pos^ than in COV^neg^ patients. These conjugates seem to have proinflammatory effects in autoimmune neuroinflammation (38). Further research is needed to elucidate the interplay between T_H_ cells and platelets, explaining its relevance for COVID-19.

Granularity of B cells was lower in COV^pos^ than COV^neg^, potentially indicating release of synthesized proteins—presumably immunoglobulins—from B cells surpasses synthesis within the cell. Similarly, granularity of CD8^pos^ T_cyt_ lymphocytes was lower in COV^non-surv^ than COV^surv^, also potentially reflecting a predominant degranulation in these patients, who died from COVID-19 (39). The lower relative lymphocyte abundance and higher leukocyte count in those patients who died confirm previous findings (40).

### The SOFA Score Correlates With Platelet Hyperreactivity and Inflammatory Markers in COV^pos^ But Not in COV^neg^ Patients—Clinical Implications

A positive correlation was observed between the SOFA score as marker for disease severity and CRP, leukocytes, and markers of platelet hyperreactivity in patients with COVID-19 only but not in those with acute respiratory disease of other reason. The peak CRP level correlated with AA- and TRAP-induced platelet aggregation and IL-6 with MPV, reflecting an interplay between platelet hyperreactivity and inflammation. Importantly, none of these relations could be demonstrated in COV^neg^ patients.

Since platelet hyperreactivity contributes to worse clinical outcome in COVID-19, it is tempting to speculate that antiplatelet-directed therapies would improve the clinical prognosis. In a pandemic retrospective analysis, aspirin administration in patients with COVID-19 reduced the risk for mechanical ventilation, ICU admission, and in-hospital mortality (41). Within the RECOVERY trial, the aspirin group did show a significant reduction in hospital stay duration, thromboembolic events, and percentage of patients who had been discharged alive compared with best medical care in COVID-19 (42). These clinical data are in line with our experimental findings and highlight the clinical impact of platelet hyperreactivity on the clinical outcome, pointing to the importance of antithrombotic therapy in COVID-19.

## Conclusion

In moderate-to-severe COVID-19, but not in other respiratory diseases, we found features of platelet hyperreactivity to be relevant for the disease severity of the patients. Our data suggest that platelet hyperreactivity together with a heightened inflammation contributes to a worse clinical outcome in patients with COVID-19, thereby pointing to the importance of antithrombotic therapy for reducing disease severity. Further clinical investigations are warranted to make use of the above-described targets.

## Data Availability Statement

The raw data supporting the conclusions of this article will be made available by the authors, without undue reservation.

## Ethics Statement

The studies involving human participants were reviewed and approved by Ethikkommission der Charité - Universitätsmedizin Berlin. The patients/participants provided their written informed consent to participate in this study.

## Author Contributions

Conception and design: KJ, UR-K, AH, and NK. Development of methodology: KJ, UR-K, and NK. Sample collection: A-CW, LR, AR, and AH. Acquisition of data: KJ, LR, MP, AR, and AA. Analysis and interpretation of data: KJ, UR-K, and NK. Writing: KJ. Review of the manuscript: UR-K, NK, MP, JF, A-CW, AR, AH, and UL. All authors contributed to the article and approved the submitted version.

## Funding

AH is participant in the BIH-Charité Advanced Clinician Scientist Pilotprogram, and JF is participant in the BIH-Charité Clinician Scientist both funded by the Charité - Universitätsmedizin Berlin and the Berlin Institute of Health. The study was supported by a research grant within the BIH & MDC Focus Area Translational Vascular Biomedicine.

## Conflict of Interest

The authors declare that the research was conducted in the absence of any commercial or financial relationships that could be construed as a potential conflict of interest.

## Publisher’s Note

All claims expressed in this article are solely those of the authors and do not necessarily represent those of their affiliated organizations, or those of the publisher, the editors and the reviewers. Any product that may be evaluated in this article, or claim that may be made by its manufacturer, is not guaranteed or endorsed by the publisher.
