# Supplementary material for: Disease Severity in Moderate-to-Severe COVID-19 Is Associated With Platelet Hyperreactivity and Innate Immune Activation
Source: Front Immunol. 2022 Mar 11;13:844701. doi: 10.3389/fimmu.2022.844701 (PMC8963244; doi:10.3389/fimmu.2022.844701)
Supplement: Supplementary file 1 [file DataSheet_1.pdf]

frontiers

Online Supplement

Disease severity in moderate-to-severe COVID-19 is associated with platelet hyperreactivity and innate immune activation

Kai Jakobs[1,2], Leander Reinshagen[1,2], Marianna Puccini[1,2], Julian Friebel[1,2,3], Anne-Christin Beatrice Wilde[4], Ayman Alsheik[1], Andi Rroku[1,2], Ulf Landmesser[1,2,3], Arash Haghikia[1,2,3], Nicolle Kränkel[1,2,†], Ursula Rauch-Kröhnert[1,2,†]

[1] Charité University Medicine Berlin, Department of Cardiology, Campus Benjamin Franklin, Berlin, Germany

[2] DZHK (German Centre for Cardiovascular Research), Partner Site Berlin, Berlin, Germany

[3] Berlin Institute of Health (BIH), Berlin, Germany

[4] Charité University Medicine Berlin, Department of Hepatology and Gastroenterology, Campus Virchow, Berlin, Germany.

[†] These authors have contributed equally to this work and share senior authorship

*** Correspondence:**

Ursula Rauch-Kröhnert, Charité - University Medicine Berlin, Berlin, Germany, ursula.rauch@charite.de

Nicolle Kränkel, Charité - University Medicine Berlin, Berlin, Germany, nicolle.kraenkel@charite.de

1 Supplemental Figures

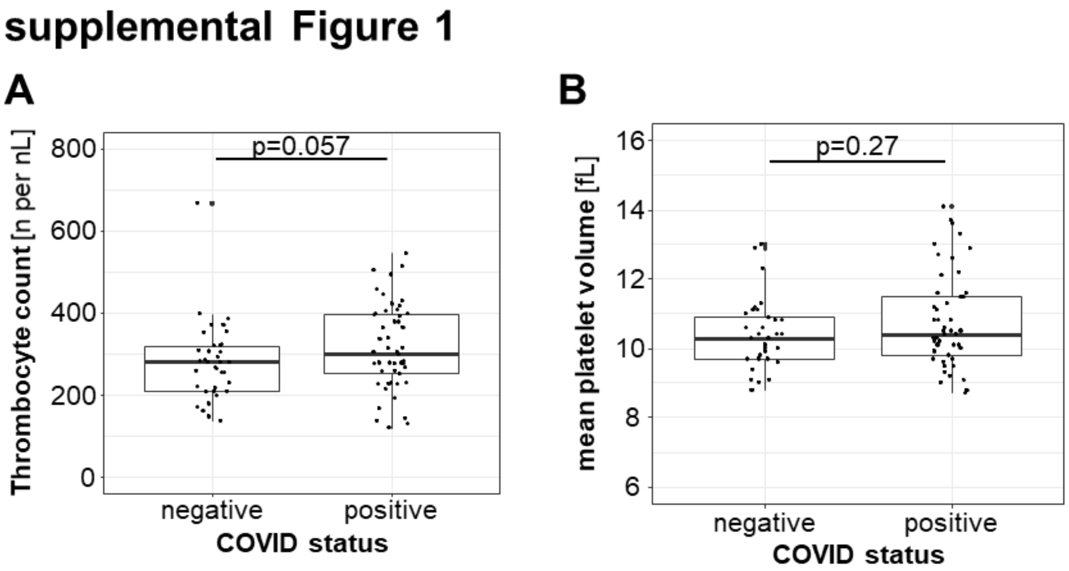

Supplemental Figure 1: No significant differences between COV^{pos} and COV^{neg} were observed for platelet count (**A**) or MPV (**B**).

supplemental Figure 2

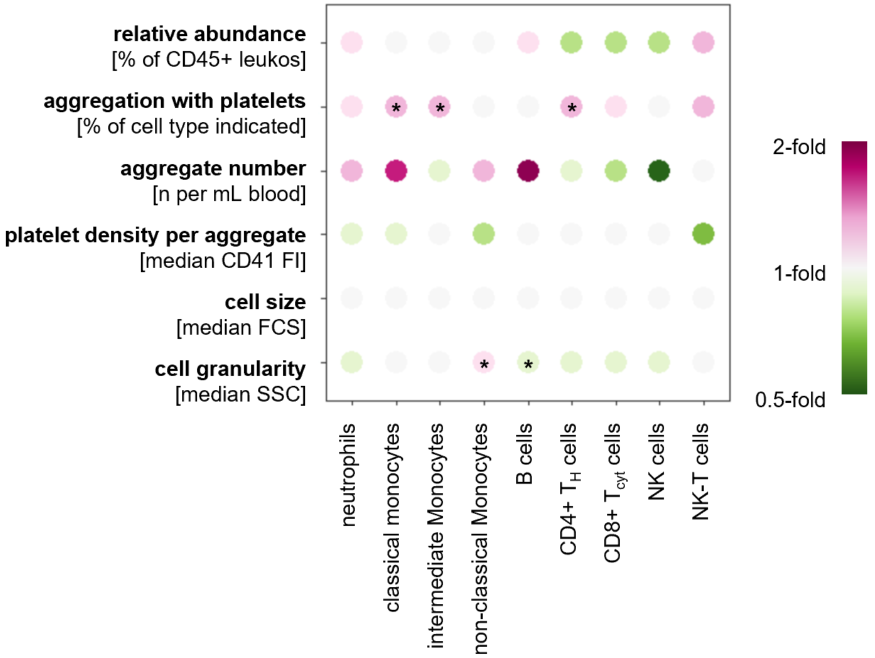

Supplemental Figure 2: Bubble plot depicting differences in characteristics of main leukocyte subtypes in COV^{pos} patients as compared to COV^{neg} patients. Colors indicate level and direction of difference between groups. Parameters with significant differences between groups are marked with *.

supplemental Figure 3

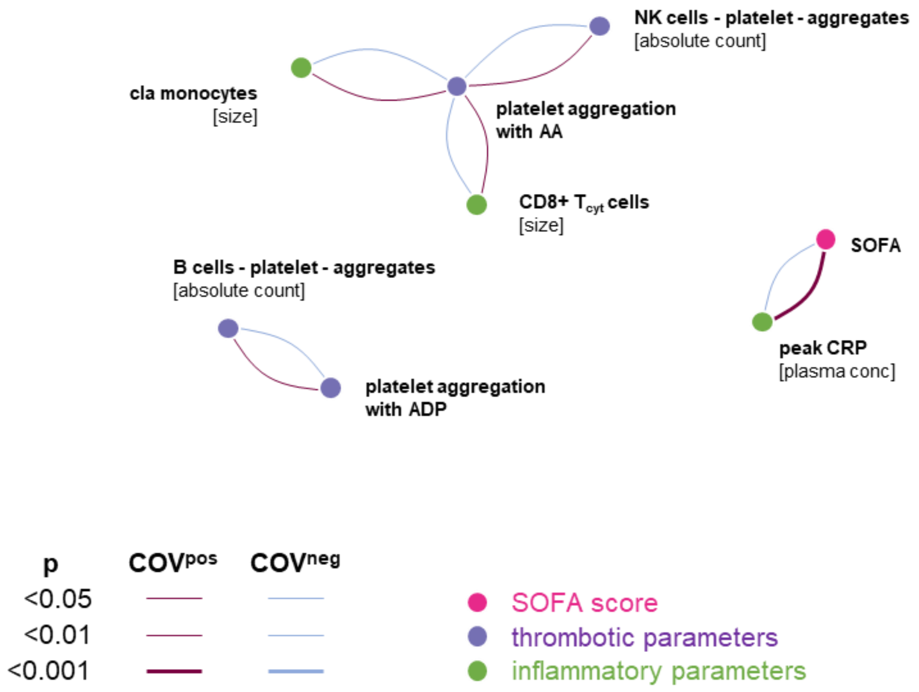

Supplemental Figure 3: Correlations observed between parameters in COV^{pos} (brown lines) and in COV^{neg} (blue lines) patients. Spearman correlations were performed and are depicted for correlations with $p<0.05$. All correlations are $r>0$. No inverse correlations ($r<0$) were observed between the same parameters in both groups. SOFA score correlates strongly with peak CRP in COV^{pos}, but only weakly in the COV^{neg} group. cla monocytes: classical monocytes, int monocytes: intermediate monocytes; ncl monocytes: non-classical monocytes, T_H: T helper cells, T_{cyt}: cytoxic T cells, NK cells: natural killer cells, peak CRP: highest CPR level during hospital stay.
